# Trends and Long-Term Mortality in Sepsis: Evidence from a Population-Based Retrospective Cohort Study of 13,994 Hospitalizations in the Abruzzo Region, Central Italy

**DOI:** 10.3390/antibiotics15060608

**Published:** 2026-06-15

**Authors:** Annalisa Marotta, Cristiano Vicenti, Camillo Odio, Jacopo Vecchiet, Marta Di Nicola, Katia Falasca

**Affiliations:** 1 Center for Excellence on Ageing and Translational Medicine (CAST), “G. d’Annunzio” University of Chieti-Pescara, 66100 Chieti, Italy; annalisa.marotta@unich.it; 2 Laboratory of Biostatistics, Department of Medical, Oral and Biotechnological Sciences, “G. d’Annunzio” University of Chieti-Pescara, 66100 Chieti, Italy; cristiano.vicenti@dxc.com (C.V.); marta.dinicola@unich.it (M.D.N.); 3 Department of Health, Information Flows and Digital Health Service, Abruzzo Region, 65121 Pescara, Italy; camillo.odio@regione.abruzzo.it; 4 Clinic of Infectious Diseases–Department of Medicine and Science of Aging, “G. d’Annunzio” University of Chieti-Pescara, 66013 Chieti, Italy; jvecchiet@unich.it

**Keywords:** sepsis, septic shock, mortality, administrative data, ICD-9-CM, epidemiology, hospitalization, healthcare costs, retrospective studies

## Abstract

Background: Sepsis remains a leading cause of morbidity, mortality, and healthcare expenditure worldwide. Despite international guidelines and diagnostic criteria, real-world variability in coding, treatment, and outcomes persist. This retrospective study analyzed 13,994 coded sepsis-related hospitalizations identified through administrative ICD-9-CM algorithms between 2016 and 2024 to evaluate the burden of sepsis, temporal trends, clinical outcomes, and healthcare costs within a regional health system. Methods: Hospitalization data across four local health authorities (ASL 201–204) over an 8-year period were analyzed. The coded sepsis cases were identified using validated ICD-9-CM-based algorithms and classified into four groups according to available microbiological coding: Gram-positive, Gram-negative, anaerobic and unspecified. Variables included patient demographics, length of stay, costs, outcomes (in-hospital and post-discharge mortality) and presence of septic shock. Comparative analyses were conducted using descriptive statistical methods and One-way ANOVA test and chi-squared tests were applied to evaluate the significance of differences. Multivariable logistic regression models were used to identify independent predictors of 6- and 12-month mortality. Results: The dataset included 13,994 coded sepsis-related hospitalizations, with the largest subgroup being ‘unspecified’ (48.0%). Among cases with specified etiology, coded anaerobic sepsis categories, though rare (0.7%), were associated with higher in-hospital mortality (45.5%) and economic burden (avg. € 8563). Mortality remained high at 6 and 12 months across all types, exceeding 50% post-discharge. Increasing age (OR ≈ 1.06 per year) and septic shock (OR ≈ 4.5–4.8) were the strongest independent predictors of mortality. Differences across microbiological groups should be interpreted cautiously given the high proportion of cases without organism-specific coding. Despite a modest reduction in mortality over time, sepsis was associated with persistently high 6- and 12-month mortality, highlighting a substantial long-term burden beyond the acute phase of illness. These findings suggest that sepsis-related hospitalizations are associated with substantial long-term mortality beyond the acute phase of illness. Discussion: These findings underscore the clinical and economic impact of sepsis in hospitalized patients, across microbiological coding categories. The high mortality rate at 6–12 months may support the need for further investigation into structured post-discharge follow-up strategies. Sepsis represents a substantial clinical and economic burden within the regional healthcare system, with persistently elevated short- and mid-term mortality. Incomplete organism-level documentation limits direct etiologic comparisons and highlights the need for improved integration between clinical, microbiological, and administrative data systems. Future research should integrate clinical variables and lab results to enable risk stratification and intervention planning.

## 1. Background

Sepsis is a life-threatening condition arising from an aberrant host response to infection [1] frequently leading to organ dysfunction and prolonged hospitalization. Despite advances in critical care, it continues to impose a substantial burden in terms of mortality and healthcare resource utilization globally [2]. Despite international guidelines and diagnostic criteria, variability persists in coding, treatment and outcomes [3,4].

Sepsis remains a major global public health challenge and is among the leading causes of preventable death worldwide. According to estimates from the Global Burden of Disease (GBD) Study, approximately 49 million sepsis cases and 11 million sepsis-related deaths occur annually, accounting for nearly one-fifth of all global deaths. Although age-standardized mortality rates have declined in many regions over recent decades, the absolute burden of sepsis remains substantial because of population ageing, increasing prevalence of chronic diseases, and improved recognition of the syndrome. Recent epidemiological studies have highlighted substantial heterogeneity in sepsis incidence, mortality, and healthcare utilization across countries and healthcare systems [5,6].

Differences in surveillance methodologies, coding practices, and clinical definitions contribute to variability in reported estimates. Population-based investigations conducted in North America, Europe, and Asia have consistently demonstrated that sepsis is associated with high short- and long-term mortality and considerable healthcare costs. Nevertheless, regional epidemiological data remain essential for understanding local disease burden and informing healthcare planning and resource allocation [2,7].

However, trends in sepsis-related mortality and healthcare utilization vary considerably across healthcare systems and patient populations [6,8]. Therefore, country-specific data are essential to understand local epidemiology and guide clinical and policy decisions. In Italy, the ICD-9-CM (International Classification of Diseases, 9th Revision, Clinical Modification) was introduced in 2000 for the coding of clinical information, particularly in the Hospital Discharge Record (SDO), with the aim of standardizing the recording of diagnoses, procedures and other health issues, facilitating health information management and data analysis in the hospital setting [9]. Although overall sepsis mortality appears to decrease with increased adherence to evidence-based care, the extent to which these improvements have benefited subgroups, such as patients with septic shock, remains unclear, particularly with differences between countries and even within different regions of the same country [10,11]. Sepsis, given its extremely high mortality and intense resource demands, justifies a dedicated epidemiological investigation. Understanding how trends in mortality, length of hospital stay (LOS), and healthcare resource utilization differ across hospitals is essential to improving the quality of clinical decision-making and developing healthcare policy [12].

## 2. Study Objectives

This study aims to evaluate the longitudinal evolution of sepsis-related outcomes and its socio-economic impact in the Abruzzo region (Italy) from 2016 to 2024. Specifically, we analyzed hospital discharge records to assess temporal trends in sepsis phenotypes, mortality rates, length of hospital stay, and healthcare costs. In addition, we aimed to identify independent predictors of 6- and 12-month mortality. Therefore the ultimate goal is to characterize temporal trends, outcomes, and healthcare burden associated with coded sepsis-related hospitalizations within a regional healthcare system.

## 3. Materials and Methods

### 3.1. Study Design and Data Source

A total of 13,994 hospital admissions across four local health authorities (ASL 201–204) with a diagnosis of sepsis were analyzed in a retrospective cohort study using routinely gathered administrative regional healthcare databases from the Abruzzo region (in Central Italy) by a descriptive population-based approach. The study period (2016–2024) was selected because it corresponds to the timeframe for which complete, standardized, and consistently coded administrative data were available within the Abruzzo regional healthcare database. Furthermore, this interval allowed the assessment of long-term temporal trends in sepsis epidemiology, healthcare utilization, and mortality outcomes over nearly a decade.

The study design, conduct, and reporting were performed according to the Strengthening the Reporting of Observational Studies in Epidemiology (STROBE) statement for observational studies (Supplementary Table S1). Administrative data referring to hospital discharges were retrieved from the regional hospital discharge database, and sepsis cases were classified according to ICD-9-CM codes. The database includes detailed patient information, including demographics (age and sex), hospital admissions and discharges, diagnoses, treatments, and procedures, making it a valuable resource for epidemiological research. The economic value of the analyzed hospitalizations was also assessed. The list of ICD-9-CM codes used in this study are provided in Supplementary Table S2.

### 3.2. Cohort Selection

The initial cohort included all hospitalizations (ordinary, day hospital, other) with sepsis (Supplementary Table S2) reported in the discharge diagnosis fields of adult patients (aged ≥ 16 years) admitted to hospitals of the four Local Health Authorities (ASL) of the Abruzzo region (L’Aquila 01, Chieti 02, Pescara 03, and Teramo 04) between September 2016 and December 2024. Patients aged ≥ 16 years were considered adults according to the organizational structure of the regional healthcare system, in which individuals from this age onward are generally managed within adult hospital care pathways.

Each hospitalization was treated as a separate unit of analysis; therefore, multiple admissions from the same patient were retained if sepsis was consistently present in at least one of the diagnoses. This approach was adopted because the primary objective of the study was to evaluate the burden of sepsis-related hospitalizations on the regional healthcare system rather than patient-level incidence. Nevertheless, repeated admissions from the same individual may have contributed to an overrepresentation of patients with recurrent or more severe disease. For each case, we collected information on the type of hospitalization regimen (ordinary, day hospital, other), hospital unit, length of stay, discharge status (discharge, death, or transfer to another facility) and clinical outcome. In this cohort, DH admissions mainly represented hemodynamically stable patients receiving continuation of antimicrobial therapy and post-acute monitoring. During part of the study period, one participating Local Health Authority implemented a structured OPAT (Outpatient Parenteral Antimicrobial Therapy) pathway for clinically stable patients requiring continuation of intravenous antimicrobial therapy after acute hospitalization.

Hospital admissions with a diagnosis of sepsis were identified using ICD-9-CM codes according to validated algorithms for administrative databases (Angus and Martin criteria), including both explicit sepsis codes and combinations of infection and organ dysfunction codes. Cases were identified for epidemiological surveillance purposes within administrative healthcare databases and were not clinically adjudicated according to Sepsis-3 criteria. The complete list of ICD-9-CM codes used for case identification and microbiological classification, together with the corresponding ICD-10-CM categories, is provided in Supplementary Table S2. This approach allows identification of the full clinical spectrum of sepsis, including culture-negative cases.

Hospitalizations were classified into four etiological groups based on available microbiological coding: Gram-positive, Gram-negative, anaerobic, and unspecified sepsis. When organism-specific ICD-9-CM codes (e.g., *Escherichia coli*, *Klebsiella* spp., *Enterococcus* spp.) were recorded, they were retained and mapped to the corresponding microbiological group. These categories were derived from administrative ICD-9-CM coding rather than direct microbiological culture data and should therefore be interpreted as coding-based epidemiological classifications. Therefore, analyses were conducted at the microbiological group level to ensure statistical robustness and clinical interpretability.

“Unspecified sepsis” was defined as discharge records containing an explicit sepsis diagnosis (e.g., ICD-9-CM 995.91, 995.92) or infection plus organ dysfunction codes, without accompanying microorganism-specific ICD-9-CM codes enabling classification into Gram-positive, Gram-negative, or anaerobic categories. This classification reflects standard limitations of large administrative datasets.

Finally, longitudinal trends were evaluated in terms of annual case numbers and all-cause mortality at 6 and 12 months post-discharge to assess the medium-term clinical burden of sepsis within the regional healthcare system.

### 3.3. Economic Evaluation

Hospitalization costs were estimated using the Diagnosis-Related Group (DRG) reimbursement tariffs applied within the Abruzzo Regional Health System, expressed in nominal Euros (€ 2024). No discounting was applied because costs were analyzed at the hospitalization level within the observed study period and not projected over future time horizons. Costs were analyzed at the hospitalization level and reported as mean ± standard deviation. This approach reflects the direct hospital reimbursement burden from the healthcare system perspective and does not include indirect or societal costs.

### 3.4. Statistical Analysis

All analyses were performed at the hospitalization level, with each admission treated as a separate observation. Because repeated admissions from the same patient could not be fully linked across all analyses, statistical models did not include patient-level clustering adjustment, as consistent patient-level linkage was not available across all hospitalization records. This represents an important limitation. Therefore, some degree of non-independence between observations may have influenced variance estimates and *p*-values. Continuous variables were summarized as mean and standard deviation (SD) and compared across etiological type of septicemia using one-way analysis of variance (ANOVA) followed by Tukey’s post hoc test for pairwise comparisons.

Categorical variables were expressed as frequencies and percentages and compared using the chi-square test (χ^2^). For temporal analyses chi-square test for trend was additionally applied where appropriate.

Annual prevalence of sepsis was calculated as the proportion of hospitalizations with a sepsis diagnosis over all hospital admissions in the corresponding calendar year; proportions were presented with 95% confidence intervals (95% CI).

To identify independent predictors of mortality, multivariable logistic regression models were fitted. The primary outcomes were 6-month and 12-month all-cause mortality. Covariates included age (continuous), sex, presence of septic shock, type of admission, microbiological classification of sepsis, and calendar year (continuous variable). Adjusted odds ratios (ORs) with 95% confidence intervals (95% CI) were reported. The unspecified sepsis category was used as the reference group because it represented the largest and most heterogeneous coding category within the administrative database.

Given the high proportion of unspecified cases, microbiological classification was interpreted with caution. Model stability was assessed by examining coefficient estimates, confidence intervals, and multicollinearity among covariates. Categories with sparse data, particularly anaerobic sepsis, were carefully evaluated to avoid unstable estimates, and results were interpreted with caution considering the limited number of observations.

As a sensitivity analysis, multivariable logistic regression models were repeated after excluding microbiological classification in order to assess the robustness of the associations observed in the main models.

All tests were two-sided and a *p*-value < 0.05 was considered statistically significant. All statistical analyses were conducted using R software (version 4.2; http://www.r-project.org/).

## 4. Results

### 4.1. Study Population

Overall, 13,994 coded sepsis-related hospitalizations were identified (Table 1) between September 2016 and December 2024. Unspecified sepsis represented the largest group (6726; 48.0%), followed by Gram-positive and Gram-negative types accounting for 26.9% (3759) and 24.3% (3408) cases, respectively, while anaerobic sepsis was uncommon, accounting for only 0.7% (101) cases, as shown in Table 1. The high proportion of unspecified cases reflects the absence of microorganism-specific coding in nearly half of discharge records and represents an important limitation when interpreting comparisons across microbiological subgroups, particularly because the unspecified category served as the reference group in regression analyses. The overall mean age of patients was 73.0 ± 16.2 years, with significantly lower values observed in Gram-positive cases (70.8 ± 16.5 years) compared with the other categories (*p* < 0.001); see Table 1. The gender distribution showed a slight male predominance overall (53.2%), with significant differences between different sepsis categories (*p* = 0.007; Table 1).

**Figure 1:**
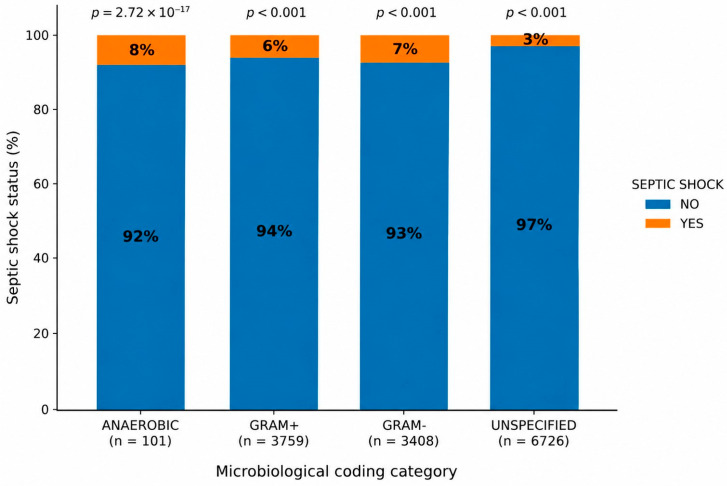
Histogram showing the distribution of septic shock outcomes across all hospitalizations stratified by types of sepsis.

In 9329 (66.7%) cases, sepsis was recorded as the primary diagnosis, with a higher proportion in Gram-negative (68.3%) and unspecified forms (68.5%) compared to the remaining etiological categories (*p* < 0.001). Septic shock was recorded in 685 (4.9%) patients, with a higher frequency in Gram-negative 250 (7.3%) and Gram-positive 230 (6.1%) compared to unspecified forms, the latter of which comprised 197 (2.9%) cases (*p* < 0.001); see Figure 1.

### 4.2. Clinical and Economic Indicators

The mean length of stay was 19.3 ± 20.9 days, with higher rates in patients with Gram-positive sepsis at 23.6 ± 23.8 days and lower rates in unspecified cases at 16.8 ± 18.1 days (*p* < 0.001). Average hospitalization costs also showed significant differences: the average overall cost was approximately 6000 ± 6716.9 euros but reached over 7200 ± 8626.0 euros for Gram-positive and Gram-negative sepsis compared to 4900 ± 4731.7 for unspecified forms (*p* < 0.001).

### 4.3. Geographic and Temporal Trends

**Figure 2:**
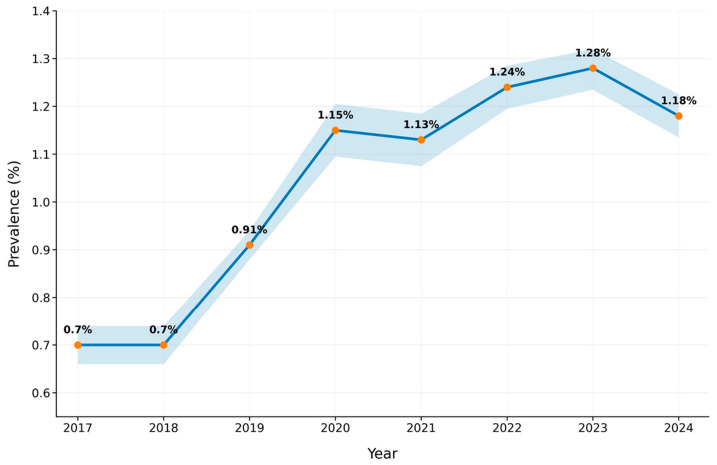
Annual prevalence of sepsis across all hospitalized patients included in the study. Line plot illustrating the proportion of hospital discharge records among all diagnosis of sepsis recorded, expressed as percentage of the total number of hospitalizations. The light blue shaded band represents 95% confidence intervals, upper and lower, respectively.

Based on hospital discharge records (SDO), the annual prevalence of sepsis among all hospital admissions ranged from 0.70% in 2016–2018 to a peak of 1.28% in 2023 (Figure 2).

Most hospitalizations occurred in ASL 203 (7641; 53.3%), followed by ASL 201 (2647; 18.9%), ASL 202 (2086; 14.9%), and ASL 204 (1800; 12.9%), *p* < 0.001. Each patient was linked to the ASL of residence showing a similar distribution with a significant proportion of patients (760; 5.4%) arriving from outside the Abruzzo region or from abroad.

Over the years, a progressive increase in the number of hospitalizations has been observed, with a peak in 2023 of 2180 (15.6%) cases and a slight decrease in 2024 with 2001 (14.3%) cases; see Table 2. A statistically significant difference was observed in the distribution of different types of sepsis over the years (*p* < 0.001).

Overall, 12,234 (89.1%) hospitalizations occurred in day hospital, while 1468 (10.7%) were ordinary inpatient admissions, with significant difference between different types of sepsis (*p* < 0.001). Analyzing hospitalizations by ASL and type of hospitalization, day hospital was predominant in all areas, with percentages ranging between 78% and 95%, whereas ordinary hospitalization was more frequent in ASL 204 (20.4%) than in other areas (*p* < 0.001).

Regarding discharges, the most common method was returning home without any additional care (5673; 40.5%), followed by death in hospital (3855; 27.5%). This latter event showed substantial variations: in-hospital mortality was particularly high in anaerobic sepsis (46; 45.5%) and in unspecified forms (2140; 31.8%) cases, respectively (*p* < 0.001).

The most frequently reported concomitant diagnoses among sepsis hospitalizations included acute respiratory failure (623; 4.5%) and viral pneumonia (300; 2.1%).

### 4.4. Outcomes

Among the overall cohort of 13,994 sepsis-related hospitalizations, follow-up information for post-discharge mortality analyses was available for 12,246 cases. Within this subgroup, 6049 (49.5%) patients died within 6 months after discharge. Cases without complete follow-up information were excluded from longitudinal mortality analyses. In subgroup analyses by microbiological classification, six-month mortality was highest among anaerobic infections (61; 65.6%) and lowest among Gram-negative infections (1324; 44.2%; *p* < 0.001). By 12 months, overall mortality rose to 53.5%, mirroring this distribution across etiologic groups (Table 3).

A significant association was observed between septic shock and mortality, both in-hospital and during the follow-up. In-hospital mortality occurred in 475 (69.3%) patients with septic shock compared with 3380 (25.4%) patients without (*p* < 0.001). At six months, mortality occurred in 472 (77.8%) patients with septic shock versus 5586 (45.9%) patients without it (*p* < 0.001); and at twelve months it further increased to 483 (81.6%) patients with septic shock and 6071 (52.1%) without (*p* < 0.001).

In multivariable analysis, increasing age and the presence of septic shock were the strongest independent predictors of both 6-month and 12-month mortality. Male sex was associated with a modest increase in mortality risk. Hospitalizations classified within the anaerobic coding category showed higher recorded mortality, whereas hospitalizations classified within the Gram-negative coding category showed a lower recorded mortality compared with the unspecified coding category. No significant temporal trend was observed for 6-month mortality, while a modest reduction over time was observed for 12-month mortality (Table 4a,b). These findings remained consistent after adjustment for key demographic and clinical variables, supporting the robustness of the observed associations.

The results were materially unchanged in sensitivity analyses excluding microbiological classification, with age and septic shock remaining the strongest independent predictors of both 6- and 12-month mortality and with no relevant changes in the magnitude or direction of the associations.

## 5. Discussion

The analysis of hospital admissions for sepsis in the Abruzzo region, Italy, reveals a scenario of substantial clinical, organizational and economic impact. Sepsis remains a condition associated with very high mortality, with more than one quarter of patients dying during hospitalization and rates exceeding 50% within one year after discharge.

In analyses restricted to hospitalizations with microorganism-specific administrative coding, the anaerobic coding category showed higher recorded mortality. However, nearly half of the cohort lacked organism-level specification, which may have reduced the reliability and interpretability of comparisons across microbiological groups. Importantly, microbiological subgroup classifications were based on administrative diagnostic coding rather than direct microbiological culture results. Therefore, these categories should not be interpreted as microbiologically confirmed bloodstream infections but rather as epidemiological coding groups derived from routinely collected healthcare data. In-hospital mortality in inadequately treated anaerobic bacteremia can reach 55%, compared to approximately 18% in cases treated promptly and effectively [13,14,15]. An analysis conducted between 2005 and 2014 showed that 30-day mortality is 38.1% in clindamycin-resistant cases, compared to 83% in susceptible strains [16,17]. Furthermore, it emerged that the progression to septic shock is strongly associated with increased mortality, a finding already widely highlighted in the literature and confirmed in this study [18].

Our findings suggest that, despite improvements in short-term outcomes, sepsis remains associated with a persistently high risk of mortality beyond hospital discharge, supporting the concept of sepsis as a condition with long-term clinical consequences rather than an isolated acute event. These findings have important implications for healthcare planning, as they highlight the need to move beyond hospital-centered care and address the long-term trajectory of sepsis survivors [19].

The present findings should be interpreted within the broader international epidemiological context of sepsis. Previous studies have shown that estimates derived from administrative databases may differ considerably from those obtained using clinically adjudicated surveillance systems. In particular, Rhee et al. demonstrated that coding practices may substantially influence apparent incidence trends, while Fleischmann et al. and Fleischmann-Struzek et al. reported considerable international variability in incidence and mortality estimates [2,5,6,7].

Despite these methodological differences, studies conducted in Europe, North America, and Asia consistently report that sepsis remains associated with substantial mortality, prolonged healthcare utilization, and significant economic burden. Recent investigations from the United Kingdom, France, Germany, Switzerland, and Japan have similarly documented persistently elevated mortality among sepsis survivors beyond hospital discharge. Although direct comparisons are limited by differences in study design, case definitions, and healthcare organization, our findings are broadly consistent with this body of evidence and support the growing recognition of sepsis as a condition associated with long-term health consequences rather than solely an acute infectious episode [17,20,21].

Evidence from low- and middle-income countries (LMICs) further highlights the magnitude of the problem, with mortality rates often exceeding those reported in high-income settings, likely reflecting differences in healthcare infrastructure, access to critical care, microbiological diagnostic capacity, and antimicrobial resistance patterns. These observations reinforce the importance of regional epidemiological studies while emphasizing the global relevance of sepsis as a major public health challenge [2,5,22,23].

The patient profile in this retrospective observation study is characterized by advanced age (mean age 73 years), male predominance, and frequent association with severe concomitant conditions such as acute respiratory failure, pneumonia, or septic shock. This reflects the high clinical complexity and vulnerability of the affected population, mainly elderly and frail, according to the literature data [24,25,26].

From an organizational and economic perspective, the impact is equally significant: the average length of stay exceeds 19 days, reaching more than 23 days in Gram-positive cases, while costs average around € 6000.00 per admission, rising above € 7000.00 in the most complex cases. This translates into substantial healthcare resource use, concentrated in specific Local Health Authorities, with admissions rising over recent years. The resulting pressure on hospital services is especially marked in Abruzzo, a region facing significant financial and healthcare problems. According to a retrospective Italian study (2016–2020), in-hospital mortality for sepsis is 19%, accompanied by a mean length of stay of 18 days and average costs of € 7508 in hospital wards. Non-ICU, up to € 15,830 in ICU, and € 10,279 in cases of septic shock [27]. In Wales, between 2006 and 2018, hospitalizations for sepsis increased from 1548 to 8708; the average cost per episode was approximately £ 7270, and 3-year mortality reached 55.2%, despite a decrease in in-hospital mortality (from 40.5% to 19.5%) [28].

High day hospital utilization and discharges home without additional support suggest a subset of patients with ongoing vulnerability and a substantial risk of mid-term mortality. This raises questions about whether care in day hospital settings provides optimal evaluation, monitoring, and interventions to reduce long-term mortality. These findings may suggest differences in patient management and follow-up between DH and ordinary hospitalization settings; however, further studies including detailed clinical variables are needed before drawing definitive conclusions regarding quality of care. Importantly, the high proportion of DH admissions may have influenced estimates of incidence, healthcare utilization, costs, and mortality because administrative sepsis-related coding may persist during post-acute care pathways and follow-up management rather than reflecting exclusively newly occurring acute sepsis episodes. Therefore, the reported hospitalization counts should not be interpreted as incidence estimates of unique patients with sepsis. Because analyses were conducted at the hospitalization level and repeated admissions may have been counted more than once, re-admissions may have contributed to the observed healthcare utilization, temporal trends, and overall burden associated with sepsis-related hospitalizations. In addition, during part of the study period, one participating Local Health Authority implemented a structured OPAT (Outpatient Parenteral Antimicrobial Therapy) pathway, recognized by the Italian Society of Infectious and Tropical Diseases (SIMIT), for clinically stable patients requiring continuation of intravenous antimicrobial therapy after acute hospitalization.

In summary, our findings are consistent with international epidemiological evidence showing that sepsis remains associated with substantial mortality, prolonged healthcare utilization, and significant economic burden across diverse healthcare systems: sepsis is not only one of the leading causes of in-hospital death but also represents a challenge in terms of clinical management, healthcare organization, and the economic sustainability of health systems [29]. Therefore, while improved microbiological identification remains clinically desirable, strengthening integration between laboratory reporting systems and administrative coding processes may be equally important. Poor long-term survival may support further investigation into structured post-discharge follow-up programs to detect complications early and reduce mortality. The persistently elevated 6- and 12-month mortality observed in our cohort is consistent with the 2026 update of the Surviving Sepsis Campaign guidelines, which highlights survivorship, recovery, re-habilitation, and longitudinal follow-up as essential components of sepsis care beyond the acute phase of illness [30].

Our results suggest a sustained clinical severity and healthcare burden associated with sepsis within the regional healthcare system. The persistently elevated 6–12 month mortality highlights the need for structured post-discharge follow-up and improved integration between clinical, microbiological, and administrative data systems. Standardizing coding practices and enhancing clinician education may reduce this diagnostic ambiguity.

Administrative data are widely used for sepsis surveillance but are sensitive to temporal and local coding practices. In a large US study, Rhee et al. [6]. reported that claims-based sepsis incidence increased over time while clinically ascertained sepsis remained relatively stable, suggesting that changes in documentation and coding can drive apparent epidemiologic trends. Therefore, some of the heterogeneity observed between hospitals/ASLs in our analyses may reflect differences in coding intensity, auditing processes, and reimbursement-related documentation. In our dataset (2016–2024), the pattern over time did not show a single abrupt step-change suggestive of an isolated coding policy shift, but rather a progressive trend. Nevertheless, we acknowledge that coding variation may contribute to both the overall increase and part of the inter-hospital differences. Importantly, the persistently high mortality and prolonged hospitalization observed among patients coded with sepsis suggest a substantial clinical burden, even if absolute rates are partly influenced by coding practices. Importantly, sepsis identification in the present study was based on administrative ICD-9-CM coding algorithms rather than direct clinical adjudication or Sepsis-3 criteria. Consequently, the findings should be interpreted within the context of coded sepsis hospitalizations used for epidemiological surveillance purposes.

### Study Limitations and Strengths

A limitation of this study is the reliance on administrative ICD-9-CM coding data, which are inherently susceptible to information bias, misclassification bias, and temporal or inter-hospital coding variability. Although validated sepsis-identification algorithms (Angus/Martin criteria) were applied and the regional SDO database is subject to official administrative quality controls, coding-based identification may not fully correspond to clinically adjudicated sepsis cases.

Nearly half of the cohort lacked microorganism-specific coding, limiting the precision and reliability of microbiological subgroup analyses and potentially introducing classification bias. Nevertheless, sensitivity analyses excluding microbiological classification yielded consistent results, suggesting that the main associations were not solely driven by pathogen-specific misclassification.

The retrospective hospitalization-based design may also have introduced selection bias. Follow-up mortality analyses were restricted to hospitalizations with available post-discharge information, and repeated admissions from the same patient may have been included more than once, potentially overestimating the burden associated with recurrent sepsis episodes. Furthermore, the administrative database did not allow a clear distinction between newly occurring acute sepsis episodes and post-acute or follow-up DH admissions related to previous sepsis-related hospitalizations. In addition, statistical analyses did not account for potential clustering effects related to repeated hospitalizations from the same individual, which may have influenced some variance estimates and *p*-values.

Furthermore, several clinically relevant variables were unavailable in the administrative database, including comorbidities, ICU admission, severity scores, antimicrobial treatment, source of infection, laboratory parameters, and detailed microbiological data. Consequently, residual confounding may have influenced some of the observed associations and limited the possibility of performing more detailed risk-adjusted analyses.

The economic evaluation was limited to DRG-based hospitalization reimbursement tariffs and did not include indirect costs, outpatient care, rehabilitation, long-term assistance, or productivity losses. Therefore, the overall healthcare and societal burden of sepsis was likely underestimated.

Furthermore, the administrative database did not include detailed clinical parameters required for Sepsis-3 classification, such as Sequential Organ Failure Assessment (SOFA) scores, laboratory markers, or direct measures of organ dysfunction.

Finally, the high proportion of cases without pathogen identification may reflect incomplete microbiological documentation, culture-negative sepsis, or coding variability and highlights the need for improved integration between microbiological, clinical, and administrative data systems.

## 6. Conclusions

This study provides a population-based overview of coded sepsis hospitalizations in the Abruzzo region over nearly a decade, highlighting the substantial clinical and healthcare burden associated with sepsis. Despite the limitations inherent to administrative coding data, persistently elevated short- and mid-term mortality rates were observed across the study period.

The findings should be interpreted within the context of retrospective administrative data and the absence of detailed clinical and laboratory information. Nevertheless, the study contributes epidemiological evidence on sepsis-related hospitalizations and long-term outcomes within a regional healthcare system.

Future studies integrating clinical, microbiological, and laboratory data are needed to improve risk stratification, better characterize sepsis phenotypes, and inform future studies aimed at developing more targeted management strategies for sepsis survivors.

## Figures and Tables

**Table 1:** Population characteristics according to different type of sepsis.

	Overall *n* = 13,994 (100.0%)	Anaerobic Sepsis *n* = 101 (0.7%)	Gram Positive Sepsis *n* = 3759 (26.9%)	Gram Negative Sepsis *n* = 3408 (24.3%)	Unspecified Sepsis *n* = 6726 (48.0%)	ANOVA Test *p*-Value
**Age, years**						
Mean (SD)	73.0 (16.2)	72.5 (17.3)	70.8 (16.5)	73.3 (15.1) *	74.0 (16.5) *	<0.001
**Gender, *n* (%)**						
Female	6547 (46.8)	47 (46.5)	1653 (44.0)	1644 (48.2)	3203 (47.6)	0.007 ^a^
Male	7446 (53.2)	54 (53.5)	2106 (56.0)	1764 (51.8)	3522 (52.4)	
**Length of stay (LOS), days**						
Mean (SD)	19.3 (20.9)	19.8 (14.9)	23.6 (23.8) ^§^	19.4 (21.9) *^§^	16.8 (18.1)	<0.001
**Healthcare cost, €**						
Mean (SD)	5998.8 (6716.9)	7291.3 (8115.0) ^§^	6784.8 (7379.4) ^§^	7260.1 (8626.0) *^§^	4900.9 (4731.7)	<0.001

^a^ Chi-squared test; * *p* < 0.05 Tukey post hoc test vs. GRAM positive sepsis; ^§^ *p* < 0.05 Tukey post hoc test vs. unspecified sepsis.

**Table 2:** Hospitalization characteristics according to different type of sepsis.

	Overall *n* = 13,994 (100.0%)	Anaerobic Sepsis *n* = 101 (0.7%)	Gram Positive Sepsis *n* = 3759 (26.9%)	Gram Negative Sepsis *n* = 3408 (24.3%)	Unspecified Sepsis *n* = 6726 (48.0%)	Chi-Squared Test *p*-Value
**Providing ASL, *n* (%)**						
201	2647 (18.9)	19 (18.8)	838 (22.3)	557 (16.3)	1233 (18.3)	<0.001
202	2086 (14.9)	20 (19.8)	508 (13.5)	938 (27.5)	620 (9.2)	
203	7461 (53.3)	56 (55.4)	1583 (42.1)	1382 (40.6)	4440 (66.0)	
204	1800 (12.9)	6 (5.9)	830 (22.1)	531 (15.6)	433 (6.4)	
**ASL of residence, *n* (%)**						
201	2542 (18.2)	20 (19.8)	815 (21.7)	519 (15.2)	1188 (17.7)	<0.001
202	2491 (17.8)	27 (26.7)	628 (16.7)	955 (28.0)	881 (13.1)	
203	6231 (44.5)	39 (38.6)	1238 (32.9)	1182 (34.7)	3772 (56.1)	
204	1970 (14.1)	9 (8.9)	842 (22.4)	567 (16.6)	552 (8.2)	
Outside region/foreign country	760 (5.4)	6 (5.9)	236 (6.3)	185 (5.4)	333 (5.0)	
**Admission year, *n* (%)**						
2016	54 (0.4)	0 (0.0)	12 (0.3)	12 (0.4)	30 (0.4)	<0.001
2017	1371 (9.8)	14 (13.9)	322 (8.6)	275 (8.1)	760 (11.3)	
2018	1144 (8.2)	12 (11.9)	274 (7.3)	301 (8.8)	557 (8.3)	
2019	1657 (11.8)	13 (12.9)	418 (11.1)	407 (11.9)	819 (12.2)	
2020	1724 (12.3)	10 (9.9)	439 (11.7)	416 (12.2)	859 (12.8)	
2021	1821 (13.0)	17 (16.8)	443 (11.8)	429 (12.6)	932 (13.9)	
2022	2042 (14.6)	13 (12.9)	583 (15.5)	435 (12.8)	1011 (15.0)	
2023	2180 (15.6)	9 (8.9)	701 (18.6)	552 (16.2)	918 (13.6)	
2024	2001 (14.3)	13 (12.9)	567 (15.1)	581 (17.0)	840 (12.5)	
**Type of admission, *n* (%)**						
Ordinary hospitalization	1468 (10.7)	8 (7.9)	488 (13.4)	276 (8.2)	696 (10.5)	<0.001
Day hospital	12,234 (89.1)	93 (92.1)	3139 (86.1)	3063 (91.5)	5939 (89.5)	
Day surgery	29 (0.2)	0 (0.0)	17 (0.5)	8 (0.2)	4 (0.1)	
**Admitting ward, *n* (%)**						
Surgical specialty wards	1025 (7.3)	22 (21.8)	317 (8.4)	323 (9.5)	363 (5.4)	<0.001
Medical specialty wards	12,968 (92.7)	79 (78.2)	3442 (91.6)	3085 (90.5)	6362 (94.6)	
**Principal diagnosis, n (%)**						
Septicemia	9329 (66.7)	58 (57.4)	2335 (62.1)	2327 (68.3)	4609 (68.5)	<0.001
Other diagnosis	4665 (33.3)	43 (42.6)	1424 (37.9)	1081 (31.7)	2117 (31.5)	
**Other diagnosis ^a^, n (%)**						
Acute and chronic respiratory failure	153 (1.1)	3 (3.0)	46 (1.2)	30 (0.9)	74 (1.1)	<0.001
Acute respiratory failure	623 (4.5)	5 (5.0)	136 (3.6)	114 (3.3)	368 (5.5)	
Septic shock	292 (2.1)	4 (4.0)	101 (2.7)	117 (3.4)	70 (1.0)	
Viral pneumoniae	300 (2.1)	3 (3.0)	86 (2.3)	57 (1.7)	154 (2.3)	
**In-hospital mortality, *n* (%)**						
Routine discharge	10,139 (72.5)	55 (54.5)	2808 (74.7)	2690 (78.9)	4586 (68.2)	<0.001
In-hospital death	3855 (27.5)	46 (45.5)	951 (25.3)	718 (21.1)	2140 (31.8)	

^a^ other diagnoses with a frequence > 150 have been reported.

**Table 3:** Death within 6 and 12 months from hospital discharges.

	Overall *n* = 12,246 (100.0%)	Anaerobic Sepsis *n* = 93 (0.7%)	Gram Positive Sepsis *n* = 3286 (26.8%)	Gram Negative Sepsis *n* = 2997 (24.5%)	Unspecified Sepsis *n* = 5870 (48.0%)	Chi-Squared Test *p*-Value
**Death within 6 months, *n* (%)**						
NO	6188 (50.5)	32 (34.4)	1642 (50.0)	1671 (55.8)	2843 (48.4)	<0.001
YES	6049 (49.5)	61 (65.6)	1642 (50.1)	1324 (44.2)	3022 (51.6)	
**Death within 12 months, *n* (%)**						
NO	5692 (46.5)	31 (33.3)	1492 (45.4)	1451 (51.4)	2628 (44.8)	<0.001
YES	6554 (53.5)	62 (66.7)	1794 (54.6)	1456 (48.6)	3242 (55.2)	

**Table 4:** (**a**) Multivariable logistic regression analysis of 6-month mortality. (**b**) Multivariable logistic regression analysis of 12-month mortality.

Variable	Adjusted OR	95% CI	*p*-Value
(**a**)			
Age (per year increase)	1.06	1.06–1.06	<0.001
Male sex (vs. female)	1.11	1.02–1.20	0.011
Septic shock (yes vs. no)	4.83	3.89–6.04	<0.001
Anaerobic sepsis (vs. unspecified)	2.05	1.27–3.37	0.004
Gram-positive sepsis (vs. unspecified)	1.08	0.98–1.19	0.105
Gram-negative sepsis (vs. unspecified)	0.70	0.63–0.77	<0.001
Year (per increase)	0.99	0.97–1.01	0.296
(**b**)			
Age (per year increase)	1.06	1.06–1.06	<0.001
Male sex (vs. female)	1.09	1.01–1.18	0.035
Septic shock (yes vs. no)	4.56	3.64–5.75	<0.001
Anaerobic sepsis (vs. unspecified)	1.84	1.14–3.04	0.015
Gram-positive sepsis (vs. unspecified)	1.15	1.04–1.26	0.005
Gram-negative sepsis (vs. unspecified)	0.72	0.66–0.80	<0.001
Year (per increase)	0.98	0.96–1.00	0.017

Abbreviations: OR, odds ratio; CI, confidence interval. Microbiological classifications were based on administrative ICD-9-CM coding categories and not on microbiologically confirmed culture data. Reference categories: female sex; no septic shock; unspecified sepsis. Odds ratios were obtained from multivariable logistic regression models.

## Data Availability

The datasets generated and/or analyzed during the current study are not publicly available due to privacy protection rules but are available from the corresponding author on reasonable request.

## Supplementary Materials

The following supporting information can be downloaded at https://www.mdpi.com/article/10.3390/antibiotics15060608/s1, Table S1: STROBE (Strengthening the Reporting of Observational Studies in Epidemiology) Checklist for the Present Study; Table S2: CD-9-CM codes used to identify sepsis cases in the administrative database and their classification into four microbiological groups, with corresponding ICD-10-CM categories.

## Abbreviations

ICD-9-CM	International Classification of Diseases, 9th Revision, Clinical Modification
SDO	Hospital Discharge Record
ASL	Local Health Authority
LOS	Length of hospital stay
DRG	Diagnosis-Related Group
SD	Standard deviation
ANOVA	One-way analysis of variance
DH	Day Hospital

## References

[1] Cao M., Wang G., Xie J. (2023). Immune dysregulation in sepsis: Experiences, lessons and perspectives. Cell Death Discov..

[2] (2025). Collaborators GBDGS. Global, regional, and national sepsis incidence and mortality, 1990–2021: A systematic analysis. Lancet Glob. Health.

[3] Shappell C.N., Klompas M., Rhee C. (2020). Surveillance Strategies for Tracking Sepsis Incidence and Outcomes. J. Infect. Dis..

[4] Mellhammar L., Wollter E., Dahlberg J., Donovan B., Olseen C.J., Wiking P.O., Rose N., Schwarzkopf D., Friedrich M., Fleischmann-Struzek C. (2023). Estimating Sepsis Incidence Using Administrative Data and Clinical Medical Record Review. JAMA Netw. Open..

[5] Rudd K.E., Johnson S.C., Agesa K.M., Shackelford K.A., Tsoi D., Kievlan D.R., Colombara D.V., Ikuta K.S., Kissoon N., Finfer S. (2020). Global, regional, and national sepsis incidence and mortality, 1990–2017: Analysis for the Global Burden of Disease Study. Lancet.

[6] Rhee C., Dantes R., Epstein L., Murphy D.J., Seymour C.W., Iwashyna T.J., Kadri S.S., Angus D.C., Danner R.L., Fiore A.E. (2017). Incidence and Trends of Sepsis in US Hospitals Using Clinical vs Claims Data, 2009–2014. JAMA.

[7] Fleischmann C., Scherag A., Adhikari N.K., Hartog C.S., Tsaganos T., Schlattmann P., Angus D.C., Reinhart K., International Forum of Acute Care Trialists (2016). Assessment of Global Incidence and Mortality of Hospital-treated Sepsis. Current Estimates and Limitations. Am. J. Respir. Crit. Care Med..

[8] Wanlumkhao W., Rattanamongkolgul D., Ekpanyaskul C. (2025). Performance of Early Sepsis Screening Tools for Timely Diagnosis and Antibiotic Stewardship in a Resource-Limited Thai Community Hospital. Antibiotics.

[9] Geraci J.M., Ashton C.M., Kuykendall D.H., Johnson M.L., Wu L. (1997). International Classification of Diseases, 9th Revision, Clinical Modification codes in discharge abstracts are poor measures of complication occurrence in medical inpatients. Med. Care.

[10] Falasca K., Vetrugno L., Borrelli P., Di Nicola M., Ucciferri C., Gambi A., Bazydlo M., Taraschi G., Vecchiet J., Maggiore S.M. (2024). Antimicrobial resistance in intensive care patients hospitalized with SEPSIS: A comparison between the COVID-19 pandemic and pre-pandemic era. Front. Med..

[11] Musat F., Paduraru D.N., Bolocan A., Palcau C.A., Bunea A.A., Ion D., Andronic O. (2025). Sepsis Burden in a Major Romanian Emergency Center-An 18-Year Retrospective Analysis of Mortality and Risk Factors. Medicina.

[12] Kamath S., Hammad Altaq H., Abdo T. (2023). Management of Sepsis and Septic Shock: What Have We Learned in the Last Two Decades?. Microorganisms.

[13] Salonen J.H., Eerola E., Meurman O. (1998). Clinical significance and outcome of anaerobic bacteremia. Clin. Infect. Dis..

[14] Hentges D.J., Baron S. (1996). Anaerobes: General Characteristics. Medical Microbiology.

[15] O’Brien J., Schrock J.W. (2025). Sepsis Presentation, Interventions, and Outcome Differences Among Men and Women in the Emergency Department. West J. Emerg. Med..

[16] Umemura T., Hamada Y., Yamagishi Y., Suematsu H., Mikamo H. (2016). Clinical characteristics associated with mortality of patients with anaerobic bacteremia. Anaerobe.

[17] Imaeda T., Oami T., Yokoyama T., Nakagawa S., Ogura H., Shime N., Umemura Y., Matsushima A., Fushimi K., Nakada T.A. (2025). Epidemiology and outcomes of septic shock in Japan: A nationwide retrospective cohort study from a medical claims database by the Japan Sepsis Alliance (JaSA) study group. Crit. Care.

[18] Edwards L., Nelmes E., Ardissino M., Qi H.L.J., Jhanji S., Antcliffe D.B., Tatham K.C. (2025). The evolution of mortality from sepsis in patients with cancer: A systematic review and meta-analysis. J. Intensive Care Soc..

[19] Shankar-Hari M., Singer M. (2017). Caring for Sepsis Patients: An Update. Crit. Care Clin..

[20] Shankar-Hari M.M., Wunsch H., Rowan K., Singer M., Rubenfeld G.D.M., Angus D.C.M. (2021). Reflections on Critical Care’s Past, Present, and Future. Crit. Care Med..

[21] Dupuis C., Bouadma L., Ruckly S., Perozziello A., Van-Gysel D., Mageau A., Mourvillier B., de Montmollin E., Bailly S., Papin G. (2020). Sepsis and septic shock in France: Incidences, outcomes and costs of care. Ann. Intensiv. Care.

[22] Machado F.R., Cavalcanti A.B., Bozza F.A., Ferreira E.M., Angotti Carrara F.S., Sousa J.L., Caixeta N., Salomao R., Angus D.C., Pontes Azevedo L.C. (2017). The epidemiology of sepsis in Brazilian intensive care units (the Sepsis PREvalence Assessment Database, SPREAD): An observational study. Lancet Infect. Dis..

[23] Dünser M.W., Noitz M., Tschoellitsch T., Bruckner M., Brunner M., Eichler B., Erblich R., Kalb S., Knöll M., Szasz J. (2024). Emergency critical care: Closing the gap between onset of critical illness and intensive care unit admission. Wien. Klin. Wochenschr..

[24] Pittet D., Thievent B., Wenzel R.P., Li N., Gurman G., Suter P.M. (1993). Importance of pre-existing co-morbidities for prognosis of septicemia in critically ill patients. Intensive Care Med..

[25] Dulger D., Ture Z., Yolcu A., Eren E.E., Sanlier N., Alp E. (2025). Sepsis in elderly patients: Investigation of prognostic factors in a secondary healthcare facility. BMC Infect. Dis..

[26] Martin G.S., Mannino D.M., Moss M. (2006). The effect of age on the development and outcome of adult sepsis. Crit. Care Med..

[27] Pipito L., Puccio R., Marrali D., Mancuso A., Gagliano M.C., Gaudiano R., Piccione M., Iaria C., Cascio A. (2024). Sepsis in Patients Hospitalized in Sicily, Italy, over the Period of 2016-2020: A Retrospective Study. J. Clin. Med..

[28] Szakmany T., Bailey R., Griffiths R., Pugh R., Hollinghurst J., Akbari A., Lyons R.A. (2025). Admissions, mortality and financial burden associated with acute hospitalisations for sepsis between 2006 and 2018: A national population-level study. J. Intensive Care Soc..

[29] Martin-Loeches I., Singer M., Leone M. (2024). Sepsis: Key insights, future directions, and immediate goals. A review and expert opinion. Intensive Care Med..

[30] Prescott H.C., Antonelli M., Alhazzani W., Møller M.H., Alshamsi F., Azevedo L.C.P., Belley-Cote E., De Waele J., Derde L., Dionne J.C. (2026). Surviving Sepsis Campaign: International guidelines for management of sepsis and septic shock 2026. Intensive Care Med..

[31] Arbuckle L.R.F. (2019). The five safes of risk-based anonymization. IEEE Secur. Priv..

[32] Zhang P., Kamel Boulos M.N. (2022). Privacy-by-Design Environments for Large-Scale Health Research and Federated Learning from Data. Int. J. Environ. Res. Public Health.

