# Supplementary material for: Trends and Long-Term Mortality in Sepsis: Evidence from a Population-Based Retrospective Cohort Study of 13,994 Hospitalizations in the Abruzzo Region, Central Italy
Source: Antibiotics (Basel). 2026 Jun 15;15(6):608. doi: 10.3390/antibiotics15060608 (PMC13295393; doi:10.3390/antibiotics15060608)
Supplement: Supplementary file 1 [file antibiotics-15-00608-s001.zip › antibiotics-4287042-supplementary.pdf]

Supplementary Table S1. STROBE (Strengthening the Reporting of Observational Studies in Epidemiology) Checklist for the Present Study

STROBE Item	Recommendation	Reported in Manuscript
**Title and Abstract**		
1a	Indicate the study's design with a commonly used term in the title or abstract	Title; Abstract
1b	Provide an informative and balanced summary of objectives, methods, results, and conclusions	Abstract
**Introduction**		
2	Explain the scientific background and rationale	Background
3	State specific objectives and hypotheses	Study Objectives
**Methods**		
4	Present key elements of study design early in the paper	Study Design and Data Source
5	Describe the setting, locations, and relevant dates	Cohort Selection
6a	Give eligibility criteria and sources/methods of selection of participants	Cohort Selection
7	Clearly define outcomes, exposures, predictors, confounders, and effect modifiers	Statistical Analysis
8	Describe data sources and methods of assessment	Study Design and Data Source
9	Describe efforts to address potential sources of bias	Study Limitations and Strengths
10	Explain how the study size was determined	Cohort Selection
11	Explain handling of quantitative variables	Statistical Analysis
12a	Describe all statistical methods	Statistical Analysis
12b	Describe methods used to examine subgroups and interactions	Statistical Analysis
12c	Explain how missing data were addressed	Statistical Analysis

STROBE Item	Recommendation	Reported in Manuscript
12d	Describe sensitivity analyses	Statistical Analysis
**Results**		
13a	Report numbers of individuals at each stage of the study	Study Population
14a	Give characteristics of study participants	Table 1
15	Report outcome events or summary measures	Outcomes; Tables 3–4
16a	Give unadjusted and adjusted estimates with precision	Tables 4a and 4b
17	Report other analyses performed	Sensitivity Analysis
**Discussion**		
18	Summarize key results with reference to objectives	Discussion
19	Discuss study limitations	Study Limitations and Strengths
20	Provide interpretation considering limitations and evidence	Discussion
21	Discuss generalizability of results	Discussion
**Other Information**		
22	Give the source of funding and role of funders	Funding Statement

Supplementary Table S2. CD-9-CM codes used to identify sepsis cases in the administrative database and their classification into four microbiological groups, with corresponding ICD-10-CM categories.

ICD-9-CM code	Disease Description	Sepsis Group	Corresponding ICD-10-CM code(s)
**038.0**	*Streptococcal sepsis*	**GRAM-POSITIVE SEPSIS**	**A40.9** (*Streptococcal sepsis*)
**038.10**	*Staphylococcal sepsis, unspecified*	**GRAM-POSITIVE SEPSIS**	**A41.0** (*Sepsis due to Staphylococcus aureus*) or **A41.2** (*Other staphylococci*)
**038.11**	*Staphylococcus aureus* sepsis	**GRAM-POSITIVE SEPSIS**	**A41.0**
**038.19**	*Other staphylococcal sepsis*	**GRAM-POSITIVE SEPSIS**	**A41.2**
**038.2**	*Pneumococcal sepsis*	**GRAM-POSITIVE SEPSIS**	**A40.3** (*Sepsis due to Streptococcus pneumoniae*)
**038.3**	*Sepsis due to anaerobes*	**ANAEROBIC SEPSIS**	**A41.4** (*Sepsis due to anaerobes*)
**038.40**	*Gram-negative sepsis, unspecified*	**GRAM-NEGATIVE SEPSIS**	**A41.5** (*Sepsis due to other Gram-negative organisms*)
**038.41**	*Hemophilus influenzae* sepsis	**GRAM-NEGATIVE SEPSIS**	**A41.3**
**038.42**	*Escherichia coli* sepsis (E. COLI)	**GRAM-NEGATIVE SEPSIS**	**A41.51** (*Sepsis due to E. coli*)
**038.43**	*Pseudomonas* sepsis	**GRAM-NEGATIVE SEPSIS**	**A41.52**
**038.44**	*Serratia* sepsis	**GRAM-NEGATIVE SEPSIS**	**A41.53**
**003.1**	*Salmonella* sepsis	**GRAM-NEGATIVE SEPSIS**	**A02.1** (*Salmonella sepsis*)
**038.9**	*Unspecified sepsis*	**UNSPECIFIED SEPSIS**	**A41.9** (*Sepsis, unspecified organism*)
**995.91**	*Sepsis (Systemic Inflammatory Response)*	**UNSPECIFIED SEPSIS**	**A41.9**
**995.92**	*Severe sepsis / Septic shock*	**UNSPECIFIED SEPSIS**	**R65.20** (*Severe sepsis*) / **R65.21** (*Septic shock*)

Unspecified sepsis includes cases without microorganism-specific ICD-9-CM codes in the discharge record.
